# Supplementary material for: Rapid estimation of cortical neuron activation thresholds by transcranial magnetic stimulation using convolutional neural networks
Source: Neuroimage. Author manuscript; Available in PMC 2023 Jul 15. (PMC10281353; doi:10.1016/j.neuroimage.2023.120184)
Supplement: 1 [file NIHMS1906430-supplement-Supplementary_Material.pdf]

Supplementary Materials

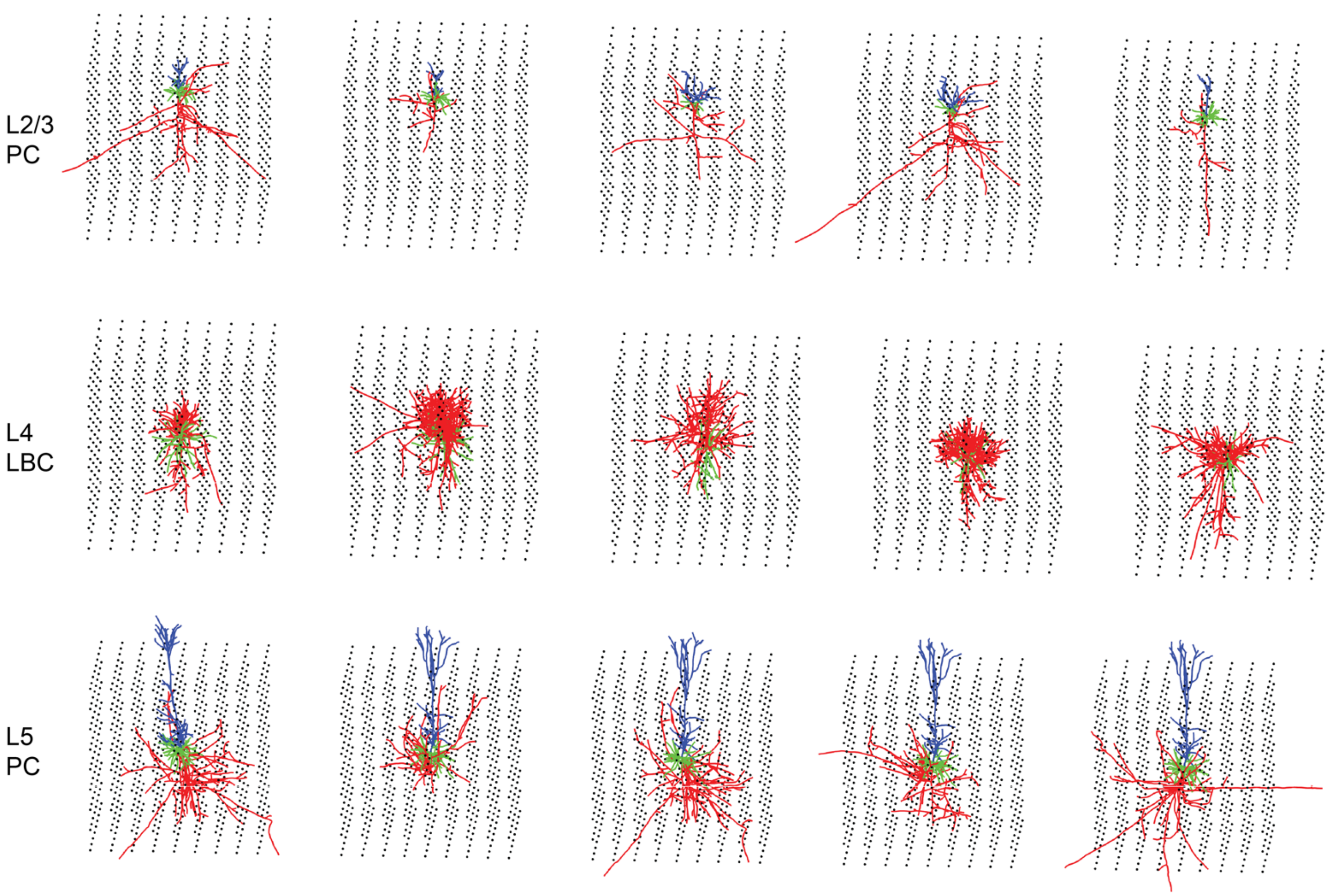

Supplementary Figure S1. E-field sampling grids for all model neurons. E-field sampling grid for **A)** L2/3 PCs with side length $l = 2$ mm shifted in z-direction by −0.49 mm; **B)** L4 LBCs with side length $l = 1.5$ mm; and **C)** L5 PCs with side length $l = 1.5$ mm. All grids shown have $N = 9$ points per dimension.

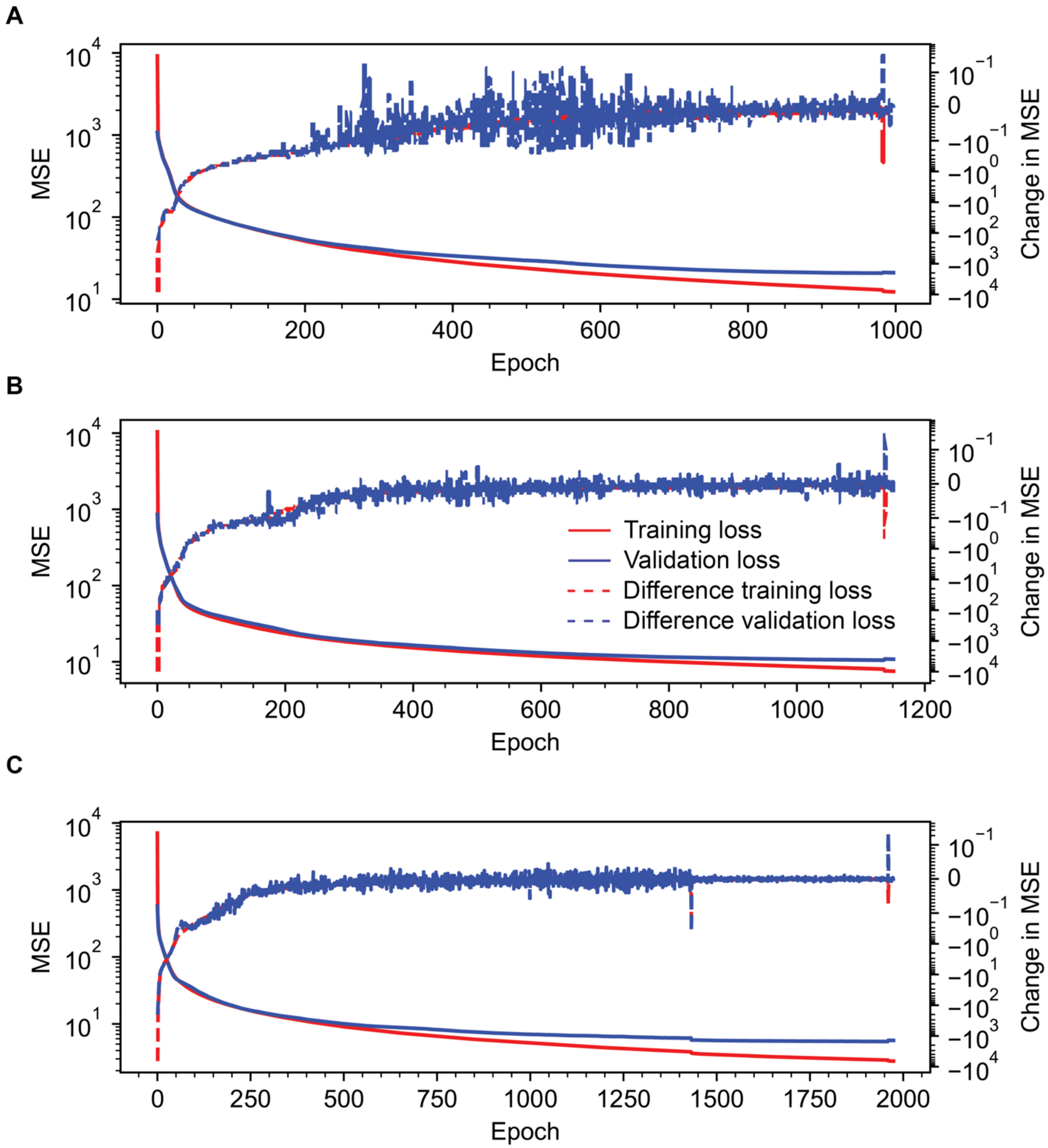

Supplementary Figure S2. Example CNN training curves. Training and validation loss, quantified as mean squared error (MSE), change in loss per training epoch for **A)** L2/3 PC (clone 1), **B)** L4 LBC (clone 1), and **C)** L5 PC (clone 1). Change in loss is plotted on symmetrical log scale with linear range between ± 10^{-1}. For reference to corresponding clone's morphology, see Supplementary Figure S1.

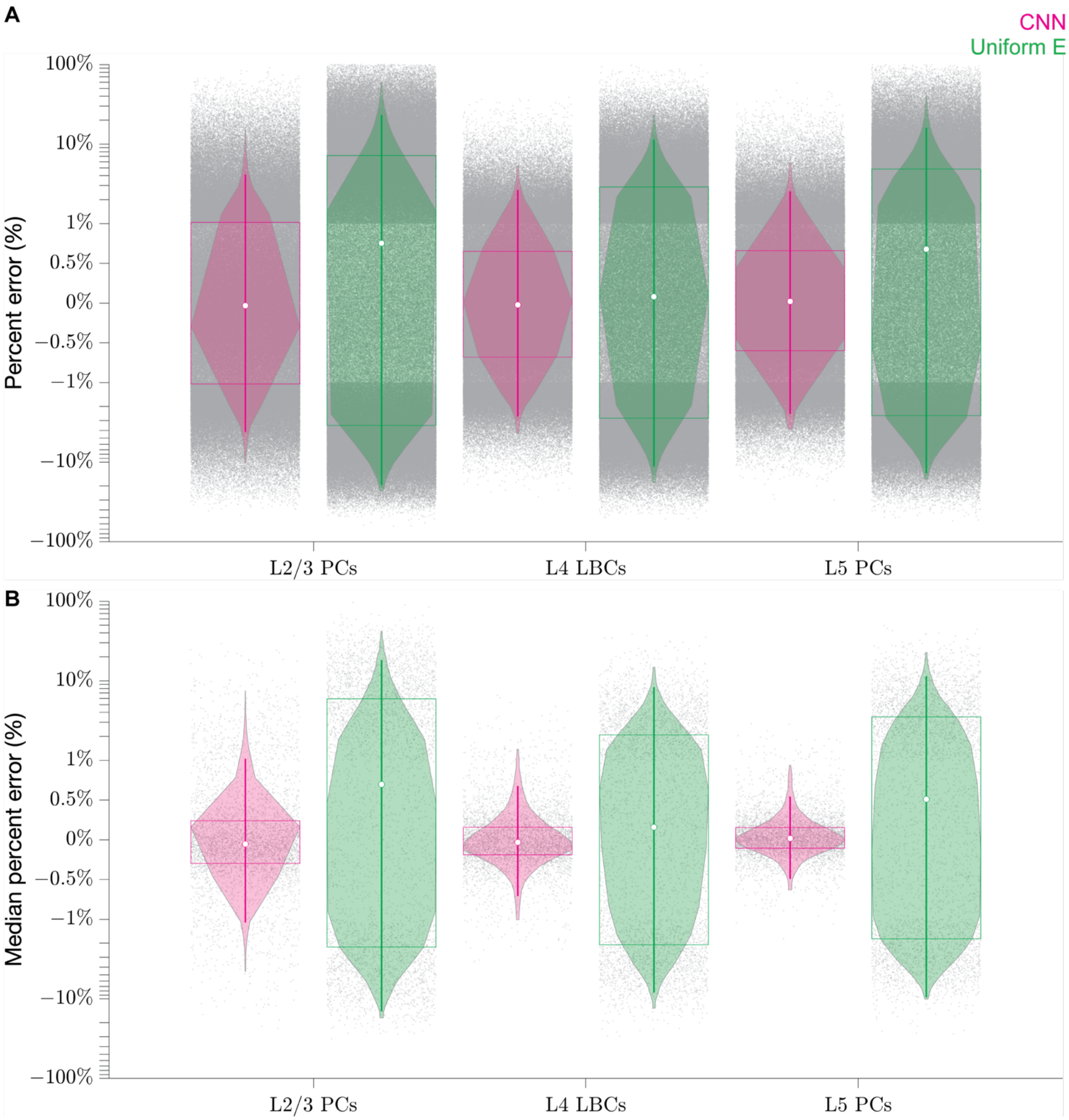

Supplementary Figure S3. Distribution of test prediction error for CNN and uniform E-field method. Distributions of **A)** percent errors of all clones and rotations and **B)** median percent errors across clones and rotations at each position shown with data points (gray); estimated probability kernel density (violin plots) spanning 98% of data; and box and whisker plots indicating median (white point), 1st and 3rd quartiles (rectangular box), and whiskers (vertical lines) extending to $1.5 \times$ interquartile range below and above 1st and 3rd quartile, respectively. Note the log-linear-log vertical axis with linear scaling between -1 and 1% and logarithmic scaling outside this range.

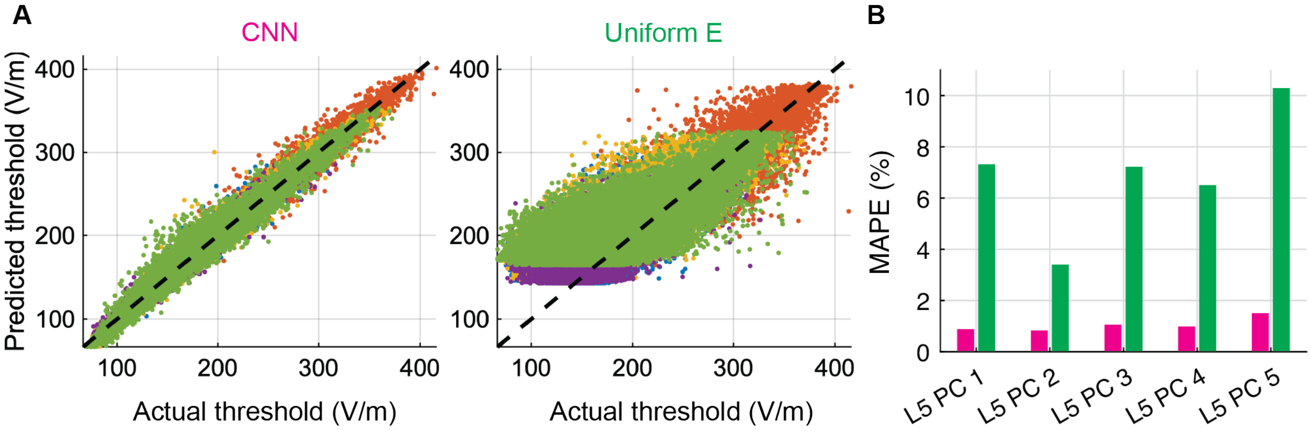

Supplementary Figure S4. CNN also predicts accurately thresholds for biphasic TMS pulse. A) Predicted threshold E-field at soma of all five L5 PC clones for MagProX100 biphasic TMS pulse by CNN (left column) and uniform E-field approximation (middle column) across entire test dataset plotted against NEURON simulation thresholds (actual). **B)** Mean absolute percent error (MAPE) on test dataset for CNN (magenta) and uniform E-field approach (green), separated by clone.

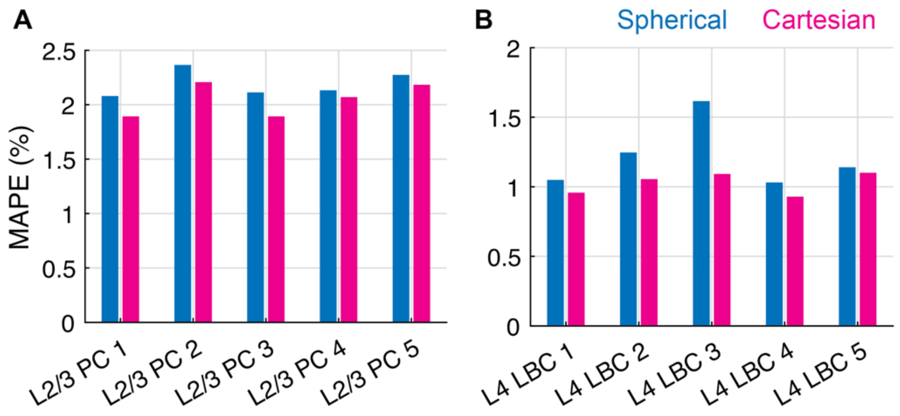

Supplementary Figure S5. Effect of E-field vector coordinate system on performance for L2/3 PCs and L4 LBCs. Mean absolute percent error (MAPE) metric on test dataset for **(A)** L2/3 PC and **(B)** L4 LBC CNNs with E-field input represented with either spherical coordinates (pre-processing described in Section 2.2.1) or Cartesian coordinates.

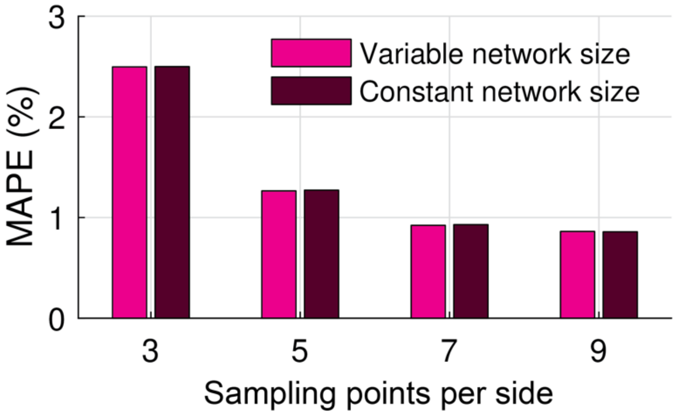

Supplementary Figure S6. CNN error increases with fewer E-field sampling points for both variable and constant network size. MAPE metric for example L5 PC (clone 1) CNN for different sampling resolutions using either variable network size, in which the architecture is modified to accommodate lower resolution inputs, or constant network size (see Section 2.2.3), in which the architecture is kept constant and the inputs are upsampled to the highest resolution ($N = 9$).

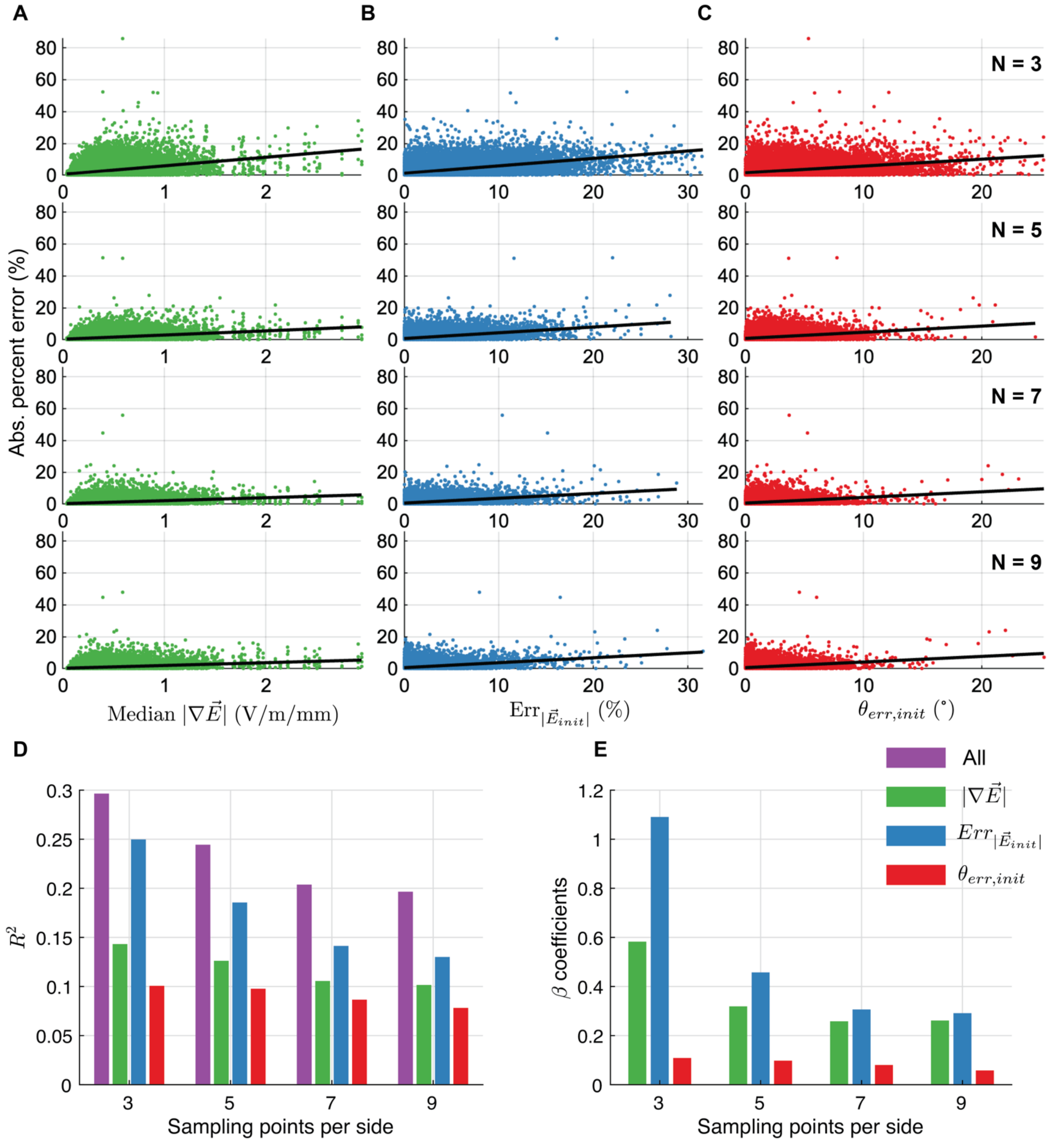

Supplementary Figure S7. CNN error correlates with E-field gradient and E-field at AP initiation site. CNN prediction errors for test dataset of example L5 PC (clone 1) plotted against **A)** median magnitude of directional E-field gradient, **B)** absolute percent error of E-field magnitude at AP initiation site, and **C)** error of E-field direction at AP initiation site for $N = 3, 5, 7,$ or 9 sampling points per dimension, with single regression lines overlaid. Median E-field gradients in A) were all calculated using E-field grids with $N = 13$ sampling points per dimension. For definition of E-field magnitude and direction error metrics in B) and C), see section 2.2.2. **D)** R^2 values for multiple linear regression with all metrics (purple) and

single regression with the metrics in A–C for each sampling resolution. Adjusted R^2 used for the multiple linear regressions to account for the effect of adding model predictors. **E)** Standardized β coefficients for multiple linear regression with all metrics in A–C at each sampling resolution.

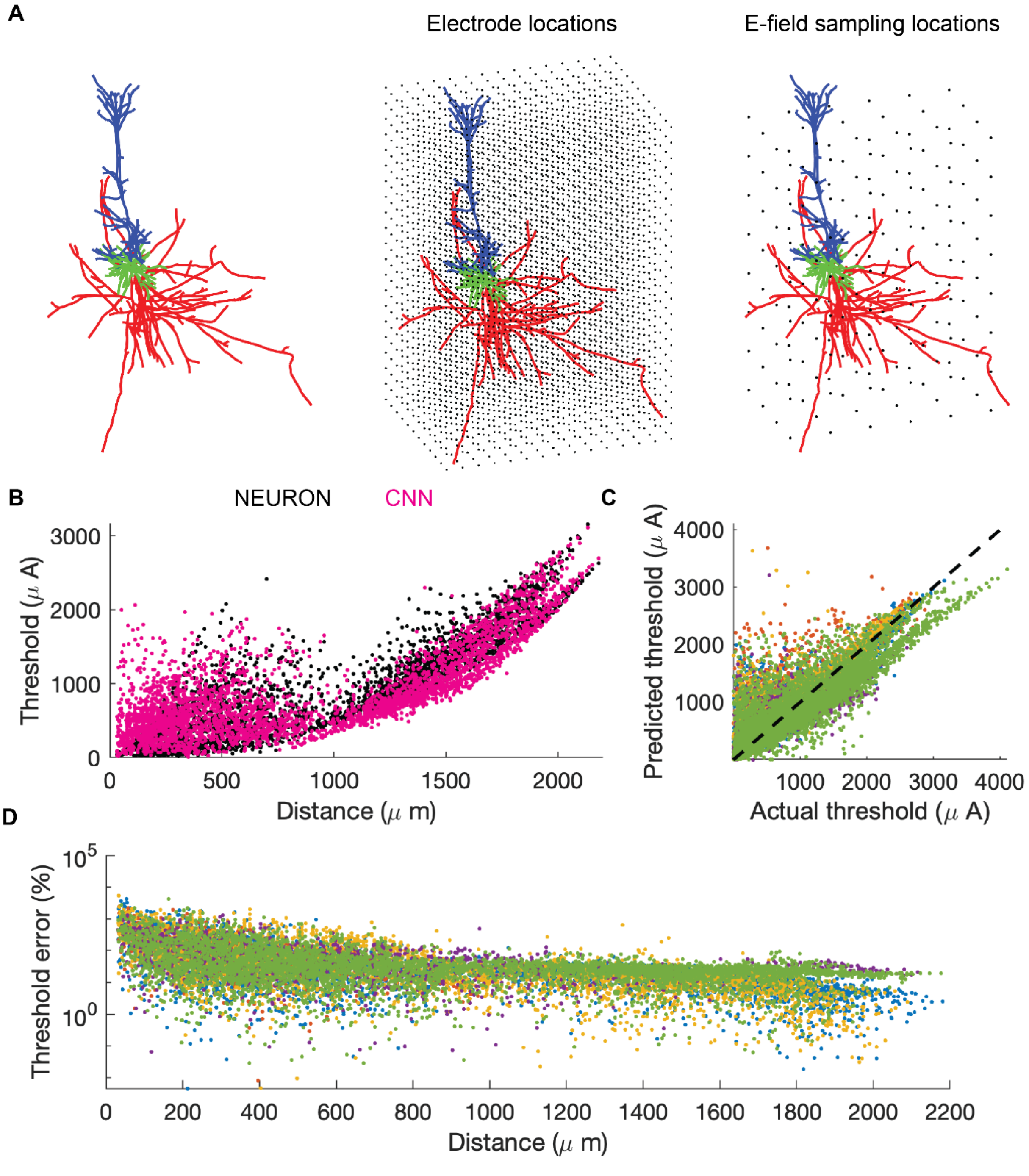

Supplementary Figure S8. CNN trained on TMS-induced E-field predicts response to point source with reduced accuracy. We modeled stimulation with a microelectrode as a point current source in a homogenous, isotropic medium with conductivity $\sigma = 0.276$ S/m, as in [19], and computed thresholds in NEURON for electrode locations throughout a 3D grid encompassing each of the L5 PC morphologies in 100 μm steps. Electrode locations within 30 μm of a neuronal compartment and eliciting dendritic activation at lowest threshold were excluded. We used the same MagProX100 monophasic TMS pulse to match the pulse waveform used with the CNNs. We estimated thresholds with the CNNs pretrained on TMS thresholds by inputting the E-field distribution generated for each electrode location, normalized to the magnitude of the center grid point. The E-field per unit current is given by $\vec{E} = 1/4\pi\sigma r^2 \hat{r}$, where r is

the electrode-to-sampling-point distance and $\hat{r}$ is the unit vector in the radial direction in spherical coordinates. **A)** Example L5 PC morphology (left) with electrode locations overlaid (middle) or E-field sampling points for CNN (right). **B)** Threshold current–distance plot for example L5 PC (clone 1) generated with NEURON simulations or the trained CNN. Each point represents the threshold for a different electrode location within the 3D grid and the distance from that electrode to the point of action potential initiation. **C)** Predicted current thresholds by CNN plotted against NEURON simulation thresholds (actual) for all five L5 PC clones. The correlations were weaker than for the TMS simulations, but were still significant, with R^2 ranging from 0.617 – 0.792 ($p < 0.001$). **D)** Threshold percent error plotted against distance for all five L5 PC clones, demonstrating lower errors for more distant electrode locations. This was likely due to the CNNs being trained on E-fields with low spatial gradients and the decrease in spatial gradient with distance from a point source.
